# Contribution to the knowledge of the Bulgarian species of the genus *Vitrea* (Gastropoda, Pristilomatidae) with the description of a new species

**DOI:** 10.3897/zookeys.396.6976

**Published:** 2014-04-02

**Authors:** Dilian Georgiev, Ivailo Dedov

**Affiliations:** 1 Department of Ecology and Environmental Conservation, University of Plovdiv, Tzar Assen Str. 24, 4000 Plovdiv, Bulgaria; 2 Institute of Biodiversity and Ecosystem Research, Bulgarian Academy of Sciences, 2 Gagarin Str., 1113 Sofia, Bulgaria

**Keywords:** New species, *Vitrea ulrichi* sp. n., Bulgaria

## Abstract

A new species of genus *Vitrea* is described: *Vitrea ulrichi* **sp. n.** is the eleventh species within the genus found in Bulgaria, and the largest representative of the Bulgarian *Vitrea*. Some critical notes on the taxonomy of the species *V. bulgarica* and *V. sturanyi* are presented. A key is provided for the determination of the species of *Vitrea* found in the country.

## Introduction

The European species belonging to the genus *Vitrea* Fitzinger, 1833 (Gastropoda: Pristilomatidae) number 56 to date, many with local and scattered distribution patterns on the continent (Welter-Schultes 2012). The shell morphology in this snail group is very important for differentiation of species as they are very diverse (Pintér 1972). In addition, the internal penis structures can be investigated but the external features of the genital organs are not of much taxonomic importance (Riedel 1992). Schileyko (2003) notes that from approximately 50 taxa in the genus known at his time, the anatomy of nearly 30 species is unknown; he also notes that the inner structure of the penis supplies the main differences between the subgenera.

There are eleven species of *Vitrea* reported in the Bulgarian fauna till now (Damjanov and Likharev 1975; Irikov et al. 2004; present study). Some of these have wider distributions and are found widespread on the European continent and/or neighboring parts of Asia or even Northern Africa, such as *Vitrea diaphana* (Studer, 1829), *Vitrea pygmaea* (O. Boettger, 1880), *Vitrea contracta* (Westerlund, 1871), and *Vitrea subrimata* (Reinhardt, 1871). The other group consists of species endemic to restricted territories situated on the Balkan Peninsula such as *Vitrea vereae* Irikov et al., 2004 and *Vitrea sturanyi* (Wagner, 1907), and some of them with distribution ranges extending also to neighboring areas such as Asia Minor (*Vitrea bulgarica* Damjanov & L. Pintér, 1969, *Vitrea neglecta* Damjanov & L. Pintér, 1969, and *Vitrea riedeli* Damjanov & L. Pintér, 1969) or the Carpathians, Central and Western Europe (Southern Germany and Northern Tirol in Austria) like *Vitrea transsylvanica* (Clessin, 1877) (Damjanov and Likharev 1975; Kerney et al. 1983; Welter-Schultes 2012; Deli and Subai 2011).

All species of *Vitrea* living in Bulgaria can well be distinguished by their shell characters (Damjanov and Likharev 1975, Irikov et al. 2004), but many aspects of their autecology are still poorly known. Some more new species in the genus can be expected. In the neighboring country of Greece, for comparison, many more species have been described, most of them representing local endemic species. Interestingly, this is not only caused by the isolation of the Greek Island, many of them are described from the continental parts of the country (Riedel 1992).

In this paper we describe a new species, *Vitrea ulrichi* sp. n. from the Stara Planina Mountain, Bulgaria, which can be distinguished from the most similar species *Vitrea kutschigi* (Walderdorff, 1864) and *Vitrea sturanyi* by its larger size, its angled shell, and very prominent shell sculpture.

## Material and methods

The specimens of the new species (and other representatives of the local malacofauna) were collected by hand and with a double sieve system (1×1 and 2×2 mm).

Abbreviations used: Nw–number of whorls, H–height of shell, D–diameter of shell, Du–diameter of umbilicus, Dlw–diameter of last whorl, Dpw–diameter of penultimate whorl; SMF–“Senckenberg Forschungsinstitut und Naturmuseum”; NMNHS–“National Museum of Natural History, Sofia”.

## Results

### Vitrea ulrichi sp. n.

http://zoobank.org/6BD6CA0F-4433-4FA0-A11F-991E6F1619BB

http://species-id.net/wiki/Vitrea_ulrichi

#### Holotype.

Nw 6.25, H 2.1 mm, D 4.65 mm, Du 0.9 mm, Dlw 0.75 mm, Dpw 0.55 mm (SMF 341898).

#### Paratypes.

2 specimens (SMF 341899/2).

The remaining paratypes are stored in the collections of the authors.

#### Locus typicus.

**Figure 1.**
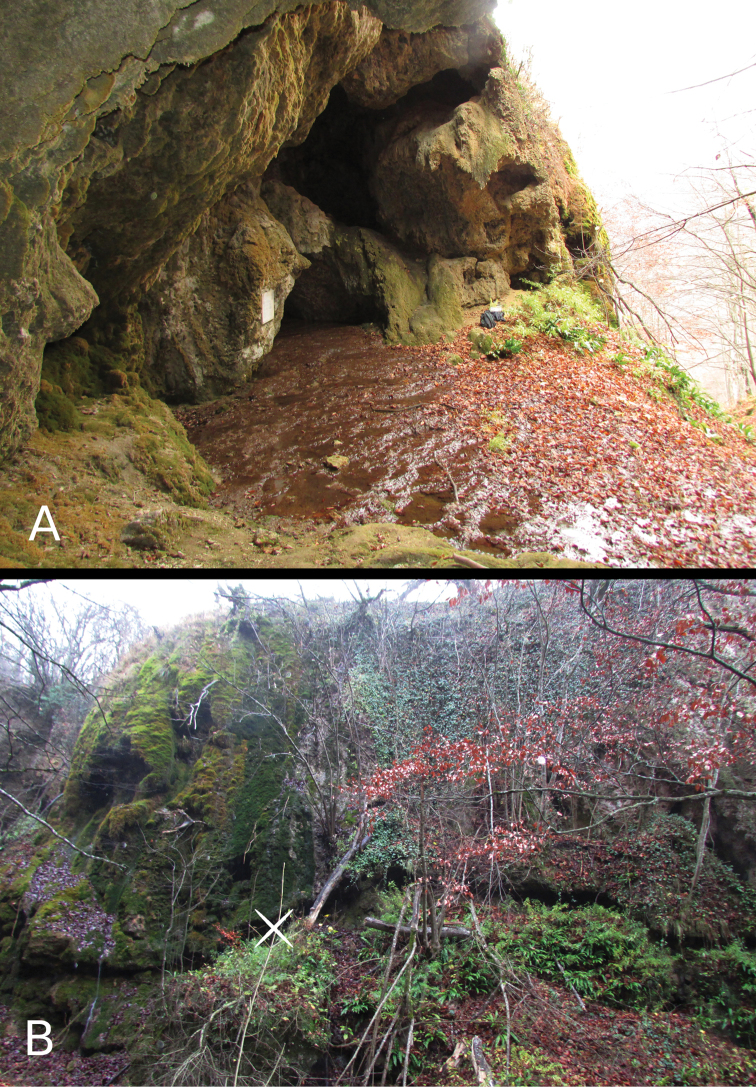
The type locality of *Vitrea ulrichi* sp. n.: the cave entrance (above) and the site of collection near the cave (below).

Surroundings of the Benkovskata Cave, near the village of Cherni Vit, Teteven town district, Stara Planina Mts, Bulgaria, 15–16.11.2013, leg. D. Georgiev, 10 adult, 5 juvenile specimens, 42°50'44.2"N, 24°10'29.8"E, 650 m (Fig. 1).

#### Etymology.

The species is named after our colleague and good friend Ulrich Schneppat (Natural History Museum, Chur, Switzerland) with gratitude for his great contribution to the knowledge of Bulgarian gastropods and for providing many literature sources, as well as for long and useful discussions with us on snails and slugs by email or around camp fires during our expeditions throughout Bulgaria.

#### Diagnosis.

Of all the *Vitrea* species reported for Bulgaria, the new species differs by its larger size, large number of whorls, and the intensely radially striated and angular shell. Considering the other European species and those distributed in the neighboring area of Asia Minor, the new species is most similar to *Vitrea kutschigi* known from Dalmatia, Serbia, Kosovo, and Macedonia, from which it differs by its coarsely striated and larger shell, higher spire, and position of the end of aperture edge on the last whorl, located at 1/3 of the last whorl in the *Vitrea ulrichi* sp. n. when compared to *Vitrea kutschigi*, where it is found on the upper side of the last whorl. The shape of the shell somewhat resembles that of *Vitrea saboorii* Neubert & Bössneck, 2013, but *Vitrea ulrichi* is bigger and has wider umbilicus.

#### Description.

**Figure 2.**
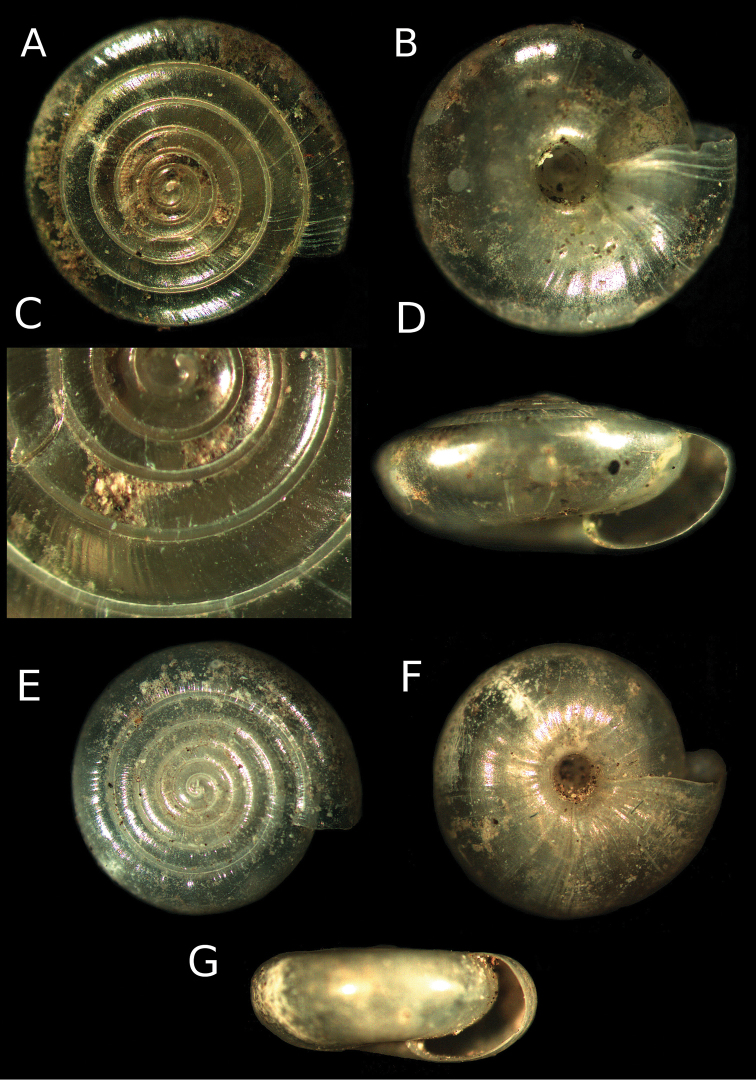
**A–D** *Vitrea ulrichi* sp. n. Shell of the holotype with view of the embryonic protoconch **E–G** *Vitrea kutschigi* from Montenegro (Dedov coll. no. Mtn 366, ex. coll. P. Subai).

The shell is translucent, yellowish-white, with 6.25–7 whorls which are densely and coarsely radially striated. The spire is low, broadly conical and elevated. The last whorl is angled at its periphery. The aperture is straight, moderately wide. In funnel perspective, the upper whorls are visible inside. The umbilicus is wide with a diameter of 0.75–1.05 mm, which represents approx. 1/5 of the shell’s diameter. The diameter of the last whorl width is less than 2 × the diameter of the penultimate whorl (Dlw 0.65–0.8 mm; Dpw 0.5–0.6 mm). The height of the shell is 2–2.35 mm. According to Welter-Schultes (2012), the shell of *Vitrea kutschigi* resembles the shell of the freshwater snail *Bathyomphalus contortus* (Linnaeus, 1758), while the shell of *Vitrea ulrichi* sp. n. is lens-like (Fig. 2).

#### Notes on the ecology.

The type locality represents the surrounding area of a limestone water cave, with a small spring flowing below the cave near its entrance, providing constant air and soil moisture. The locality, where the new species was found, is a steep carbonate rock on the right side of the cave, densely covered with broad leaf detritus, mainly from *Fagus sylvatica*. The area is occupied by *Fagus sylvatica* and *Carpinus betulus* trees and bushes, as well as mosses and ferns (mostly *Asplenium scolopendrium*) covering the rocks (Fig. 1).

The terrestrial malacofauna diversity at the type locality was very rich. There were more than 20 species of land gastropods registered, within only on a few square meters of area: *Carychium tridentatum* (Risso, 1826), *Agardhiella* cf. *pirotana* Subai, 2011, *Vallonia pulchella* (O. F. Müller, 1774), *Cochlicopa lubricella* (Porro, 1838), *Laciniaria* cf. *plicata* (Draparnaud, 1801), *Macedonica marginata* (Rossmässler, 1835), *Alinda wagneri* (A. J.Wagner, 1911), *Vestia ranojevici* (Pavlovic, 1912), *Euconulus fulvus* (O. F. Müller, 1774), *Vitrea diaphana* (Studer, 1829), *Vitrea transsylvanica* (Clessin, 1877), *Vitrea bulgarica* Damjanov & L. Pintér, 1969, *Vitrea contracta* (Westerlund, 1871), *Aegopinella pura* (Alder, 1830), *Oxychilus glaber* (Rossmässler, 1838), *Daudebardia brevipes* (Draparnaud, 1805), *Perforatella incarnata* (O. F. Müller, 1774), *Euomphalia strigella* (Draparnaud, 1801), *Cattania balcanica* (Kobelt, 1876), and *Cepaea vindobonensis* (Férussac, 1821).

## Discussion

After the description of this new species, the genus *Vitrea* in Bulgaria encompasses eleven species. In this number, we also include some doubtful species such as *Vitrea bulgarica* and *Vitrea sturanyi*. Due to lack of anatomical data, we are not able to confine the new species to one of the existing subgenera.

### The problem of *Vitrea bulgarica*–*Vitrea neglecta*

Damjanov and Pinter (1969) described the two species *Vitrea neglecta* (locus typicus: Bulgaria, Rhodope Mountains, some kilometers from the Bachkovski Monastery, Chaya river valley) and *Vitrea bulgarica* (locus typicus: Bulgaria, Rhodope Mountains, tributary of Chaya river between Asenovgrad and Bachkovo) in the same work.

Dedov (1998) suggested that the status of both species should be re-evaluated and that internal anatomies should be studied. Irikov (2001), after examination of shell morphology and anatomy of specimens from both type localities, concluded that *Vitrea bulgarica* and *Vitrea neglecta* were synonyms. This opinion was accepted by Welter-Schultes (2012).

**Figure 3.**
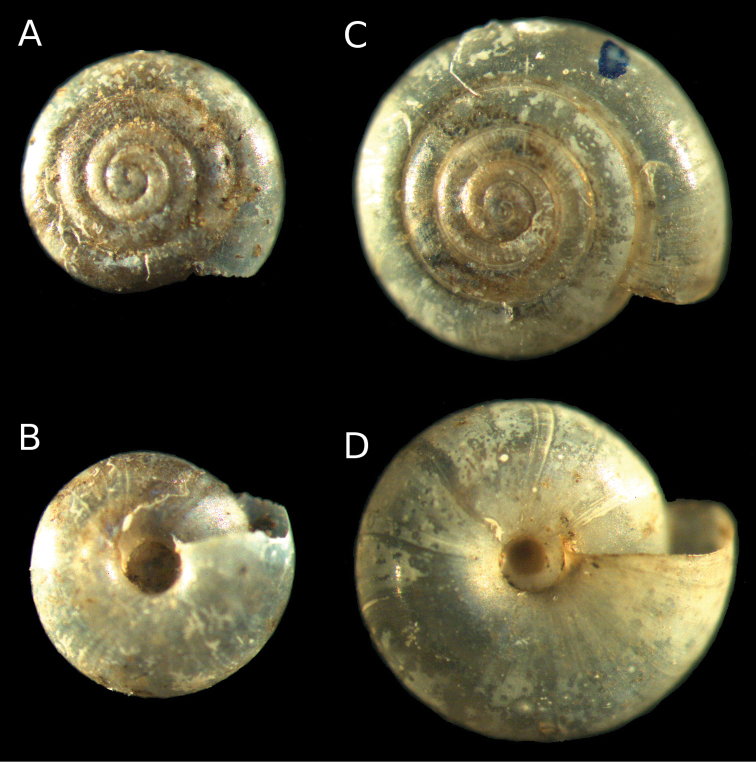
**A, B** *Vitrea neglecta* Damjanov & L. Pintér, 1969: paratype NMNHS 6704, **C, D** *Vitrea bulgarica* Damjanov & L. Pintér, 1969: holotype NMNHS 6627.

The examination of material from genus *Vitrea* stored in the NMNHS revealed the existence of the holotype of *Vitrea bulgarica* (NMNHS 6627, information from the label: Asenovgrad, 24.07.1967, leg.L. Pintér) and a paratype of *Vitrea neglecta* (NMNHS 6704, information from the label: S. of Smolyan, 11.06.1967, leg. S. Damjanov, det. L. Pintér) (Fig. 3).

After studying these specimens, we found some differences existing between *Vitrea bulgarica* and *Vitrea neglecta*, which correspond to the original descriptions of both species (Damjanov and L.Pinter 1969). In *Vitrea bulgarica*, the whorls increase faster than in *Vitrea neglecta*; the last whorl is approximately two times larger than the penultimate and the umbilicus is in form of a funnel, respectively. Moreover, in *Vitrea neglecta* the suture looks much deeper if compared to *Vitrea bulgarica*. Because of the poor quality of the shell of the paratype specimen of *Vitrea neglecta*, the radial sculpture of the shell is not clearly visible. At the same time, the more convex lower side of the shell of *Vitrea bulgarica* (Damjanov and L.Pinter 1969; Damjanov and Likharev 1975) is not clearly discernible; in addition, the correlations of the diameters of the umbilicus to diameter of the shells differs from those given by Damjanov and Likharev (1975) (*Vitrea bulgarica* Du/D = 1/9; *Vitrea neglecta* Du/D = 1/10–1/11). According our measurements, the umbilici in both species are wider than the information provided by Damjanov and Likharev (1975). The parameter of both shells are: *Vitrea bulgarica* – Nw 5.25, D 3.09, Dlw 0.72, Dpw 0.36, Du 0.48, H 1.29, Du/D = 1/6.5; *Vitrea neglecta* – Nw 4.5, D 2.19, Dlw 0.45, Dpw 0.3, Du 0.43, H 1.08, Du/D = 1/5.

Irikov’s opinion (2001) “between typical *Vitrea neglecta* and *Vitrea bulgarica* there are many intermediate forms, forming gradual series” could be interpreted as a confirmation of Riedel (1992), who commented on the difficulties recognizing apparent differences between the two species in some populations. The different forms and difficulties in determination exist also in other species of this genus (Pintér 1972, Damjanov and Likharev 1975; Riedel 1992). To our opinion, some forms considered as “intermediate” probably represented juvenile or sub-adult stages of the shell (for *Vitrea bulgarica* and *Vitrea neglecta* it means less than 4.5–5 whorls). Our observations on the shell morphology of adult specimens (4.5 whorls and more) of *Vitrea bulgarica* from western Bulgaria shows populations of typical *Vitrea bulgarica*, with variations in the border of species characters. Thus whenever we speak about intermediate forms within the genus *Vitrea* it is necessary to indicate the size of the studied species, respectively the number of their whorls.

The most important question for a correct determination of the species in genus *Vitrea* concerns the structures of the sexual system. According to Pintér (1972), the shell morphology in this genus is paramount for differentiation of species, and Riedel (1992) stated that the external features of the genital organs are not of much taxonomic importance. However, the internal structure of the penis provides information that can be used for a sub-generic distinction (Schileyko 2003). Probably this is the reason, despite their comments about the close relationship between *Vitrea bulgarica* and *Vitrea neglecta*, why Damjanov and Likharev (1975) and Riedel (1992) accepted both species as separate. The question is “how far can we rely on the structure of the sexual system in this genus when discussing closely related species?” In our opinion, the structure of the sexual system is important, but is not the single character that should form the basis of a taxonomic opinion. In this case, it is important to study the sexual systems of those specimens, who are considered to represent “border” forms. After that, the probably can be determinate more clearly as known species or intermediate forms. Without completely rejecting the conclusion of Irikov (2001) at this stage, we currently consider the problem *Vitrea bulgarica*–*Vitrea neglecta* still as open requiring more detailed studies, which are planed by the authors for the near future.

### Vitrea sturanyi

The occurrence of *Vitrea sturanyi* in Bulgaria, and even on the East Balkans, is disputable. Wagner (1907) described *Vitrea sturanyi* (as *Crystallus sturanyi* Wagner, 1907) from Bosnia, Krupa spring near Pazarich. Later, Wohlberedt (1911), Hesse (1916) and Jaeckel (1954) reported this species also for Bulgaria. Pintér (1972) challenged these records and referred them to other Bulgarian species like *Vitrea bulgarica*, *Vitrea neglecta*, *Vitrea diaphana*, *Vitrea contracta*, and even *Oxychilus hydatinus* (Rossmässler, 1838) from the family Oxychilidae. Damjanov and Likharev (1975) confirmed the species for Bulgaria from two localities in the Western Rhodope Mountains (Velingrad and Trigrad village), while Welter-Schultes (2012) negates the occurrence of this species in Bulgaria. Our shells from southwestern Bulgaria show some differences when compared to the descriptions of Damjanov and Likharev (1975)–larger diameter of the shell, a smaller number of the whorls, and much more depressed spire. It is currently not clear whether this could be intra-specific variation of *Vitrea sturanyi*, or whether this represents another new species. To clarify this problem it is needed to compare our Bulgarian populations with the type specimens from Bosnia, which is also another activity for the near future.

Summarising the current knowledge on the genus *Vitrea* in Bulgaria, we propose the following key to identify the species within the country:

**Table:** 

1	umbilicus entirely closed	2
–	umbilicus more or less open	4
2	diameter of the last whorl only slightly wider than penultimate whorl	*Vitrea diaphana diaphana*
–	diameter of the last whorl almost 3 times wider than penultimate whorl	*Vitrea transsylvanica*
4	umbilicus with minute opening, through the umbilicus internal whorls cannot be seen, whorls is 4.5–5, diameter of the shell 3.0–4.3 mm	*Vitrea subrimata*
–	umbilicus much wider, the penultimate whorls through the umbilicus could be seen	5
5	diameter of the last whorl almost 2 times wider than penultimate whorl	6
–	diameter of the last whorl less wide (1.5 time than penultimate whorl or even less)	7
6	suture deep, mouth is wider, size smaller (in 3.5–4 whorls, diameter of shell 1.4–2.1 mm, the height of shell 0.7–0.8 mm)	*Vitrea pygmaea*
–	suture shallow, the mouth is narrowed, size bigger (in 4.5–5.5 whorls, diameter of shell 2.9–3.2 mm, the height of shell 1.3–1.5 mm)	*Vitrea bulgarica*
7	umbilicus perspective, very wide (about 1/3 from shell diameter), the whorls is 3–3.5	V. verae
–	umbilicus perspective, moderately wide, 1/5 or even less from shell diameter, the whorls are 4.5 or more	8
8	umbilicus perspective, about 1/4–1/6 from shell diameter	9
–	umbilicus much narrow (about 1/12–1/14 of the shell diameter), the shell smooth, finely striated near the suture only, the bottom side of the shell rounded	*Vitrea contracta*
9	shell intensively radially striated, the number of whorls is 6.5–7, diameter of the shell big (4.65–5.3 mm), shell with angled periphery	*Vitrea ulrichi* sp. n.
–	shell smooth or finally striated, the number of whorls is smal 4.5–5.75, diameter of the less than 4.3 mm	10
10	shell smooth, the spire much conical, the umbilicus much wide (1/4–1/5 from shell diameter)	*Vitrea riedeli*
–	shell finally striated, the spire much depressed, the umbilicus much close (1/5–1/6 from shell diameter)	11
11	shell bigger (in 5 whorls diameter of the shell is 3.8–4.3 mm), umbilicus perspective-cylindrical	*Vitrea* cf. *sturanyi*
–	shell smaller (in 5 whorls diameter of the shell is 2.9–3 mm), umbilicus perspective-conical	*Vitrea neglecta*

## Supplementary Material

XML Treatment for Vitrea ulrichi
